# Ultrasound of Sternal Fracture

**DOI:** 10.5811/westjem.2015.9.28645

**Published:** 2015-12-08

**Authors:** Shadi Lahham, Jonathan Patane, Nathaniel Lane

**Affiliations:** University of California, Irvine, Department of Emergency Medicine, Irvine, California

## PRESENTATION

**Figure 1:**
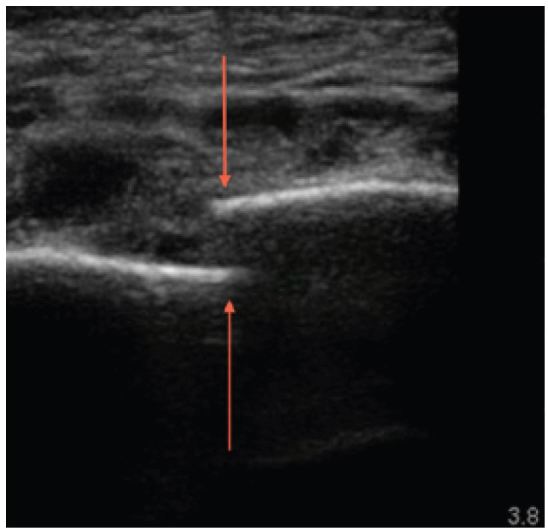
Ultrasound image of the sternum, with the red labeling the two ends of a displaced sternal fracture.

A 61-year-old female was brought in by ambulance after being the restrained driver of a head-on motor vehicle collision at 40MPH. There was positive airbag deployment and intrusion from the other vehicle. During workup, the patient complained of midline chest pain, and left chest wall pain. The patient was not in acute respiratory distress, and had the following vital signs: temperature 37°C, heart rate 84, blood pressure of 150/64, respiratory rate 18, and oxygen saturation of 97% on two liters of oxygen. On physical exam, breath sounds were heard bilaterally, with no acute cardiopulmonary issues identified. A bruise was identified on the lower abdomen, which was thought to be a potential seatbelt sign. A focused assessment with sonography for trauma was negative, and an ultrasound of additional chest and mediastinal structures was performed for the chest tenderness (Figure 1).

## DIAGNOSIS

**Figure 2:**
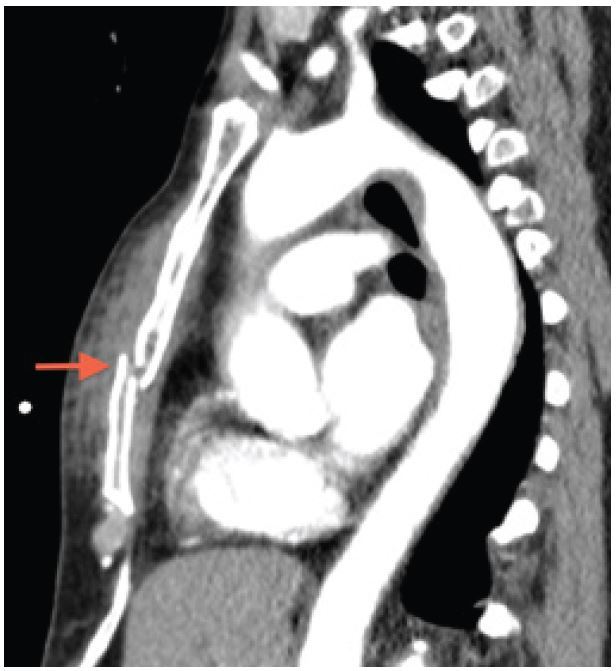
Computed tomography identifying the same displaced sternal fracture.

Sternal fracture has been observed in approximately 10% of patients with blunt chest trauma, with the most common mechanism of injury being motor vehicle accidents.^1^ Isolated sternal fractures most often have a benign course, but can rarely cause secondary cardiac injury.^2^ Patients with chest trauma typically undergo radiograph imaging in the emergency department to help rule out acute life-threatening cardiopulmonary injuries such as aortic dissection, tension pneumothorax, and cardiac tamponade, among other pathologies. Typically, these imaging techniques involve a portable chest radiograph, followed by a computed tomography (CT) of the chest if applicable.^3^ Standard AP chest radiographs have a low sensitivity for diagnosing sternal fractures, with the majority of fractures being identified by lateral chest radiograph or CT (Figure 2). Because lateral chest radiographs are typically not performed in the acute trauma workup, many sternal fractures are not diagnosed until later in the trauma evaluation.^1^,^4^

Recent studies have compared the sensitivity and specificity of chest radiographs and ultrasound in determining the presence of sternal fracture. The sensitivity and specificity of chest radiograph were 70.8% and 75.0%, respectively with ultrasound showing a sensitivity and specificity as high as 100%.^4^ Ultrasound has the advantage of increased sensitivity and specificity for diagnosing sternal fractures in comparison to chest radiographs, and avoids the excess radiation and time commitment of mobilizing patients to perform a chest CT.^4^ Ultrasound is not accurate in identifying the degree of displacement of sternal fractures, but can accurately identify related hematomas and pleural effusions.^4^
